# Avaliação Psicométrica da Prova de Título de Especialista em Cardiologia da Sociedade Brasileira de Cardiologia

**DOI:** 10.36660/abc.20220355

**Published:** 2022-11-09

**Authors:** Gustavo Eugênio Martins Marinho, José Maria Peixoto, José Knopfholz, Marcus Vinicius Santos Andrade

**Affiliations:** 1 Faculdade de Medicina Universidade José do Rosário Vellano Alfenas MG Brasil Faculdade de Medicina na Universidade José do Rosário Vellano (UNIFENAS), Alfenas , MG – Brasil; 2 Universidade José do Rosário Vellano Belo Horizonte MG Brasil Universidade José do Rosário Vellano (UNIFENAS), Belo Horizonte , MG – Brasil; 3 Programa de Mestrado Profissional em Ensino em Saúde Universidade José do Rosário Vellano Belo Horizonte MG Brasil Programa de Mestrado Profissional em Ensino em Saúde da Universidade José do Rosário Vellano (UNIFENAS-BH), Belo Horizonte , MG – Brasil; 4 Pontifícia Universidade Católica do Paraná Curitiba PR Brasil Pontifícia Universidade Católica do Paraná , Curitiba , PR – Brasil; 5 Programa de Especialização em Educação em Saúde Universidade de São Paulo São Paulo SP Brasil Programa de Especialização em Educação em Saúde da Universidade de São Paulo , São Paulo , SP – Brasil; 6 Escola Bahiana de Medicina Salvador BA Brasil Escola Bahiana de Medicina , Salvador , BA – Brasil; 7 Hospital Santa Izabel Santa Casa da Bahia Salvador BA Brasil Hospital Santa Izabel - Santa Casa da Bahia , Salvador , BA – Brasil; 8 Hospital Aliança Salvador BA Brasil Hospital Aliança , Salvador , BA – Brasil

**Keywords:** Especialização, Cardiologia, Psicometria

## Abstract

**Fundamento:**

A Sociedade Brasileira de Cardiologia promove anualmente a prova para obtenção do título de especialista em Cardiologia, sendo a Comissão Julgadora do Título de Especialista em Cardiologia responsável pela sua organização e aplicação. A análise psicométrica dos itens de uma prova, por meio da Teoria de Resposta ao Item (TRI) pode fornecer informações robustas e contribuir para o aprimoramento contínuo dessa avaliação.

**Objetivos:**

Avaliar as propriedades psicométricas da prova do Título de Especialista em Cardiologia no ano de 2019, em relação aos parâmetros da TRI.

**Métodos:**

Estudo observacional, com a análise psicométrica das 120 questões da prova realizada por 1120 (mil cento e vinte) candidatos para a obtenção do título de especialista em Cardiologia, no ano de 2019.

**Resultados:**

A análise da prova pela TRI mostrou 32,2% dos itens com poder de discriminação “alto” ou “muito alto”, 49,2% dos itens categorizados como “fácil” ou “muito fácil” e 41,5% apresentavam alta probabilidade de *acerto ao acaso* . Foram identificados 69 itens com problemas em relação aos parâmetros da TRI e, portanto, com baixo poder de avaliar a proficiência do candidato.

**Conclusões:**

A análise psicométrica da prova de título de Especialista em Cardiologia pela TRI demonstrou um alto percentual de questões fáceis, com cerca de dois terços dos itens com alta probabilidade de *acerto ao acaso.* Esses dados poderão desencadear uma série de discussões e propostas para a construção das futuras provas em cardiologia.

## Introdução

A busca pelo título de especialista tem se tornado uma constante entre os médicos brasileiros. As razões envolvem desde o ganho de conhecimento, permissão para participar de concursos, até o ingresso em cooperativas médicas no mercado de trabalho, tornando-se evidente que a titulação aumenta o prestígio do profissional e de sua especialidade.

O Título de Especialista em Cardiologia (TEC) existe na Sociedade Brasileira de Cardiologia (SBC) desde 1968, porém somente foi regulamentado pela Associação Médica Brasileira (AMB) e pelo Conselho Federal de Medicina (CFM) por meio da Resolução n. 1286/89. Nesse contexto, surge, em 1992, a Comissão Julgadora do Título de Especialista em Cardiologia (CJTEC). ^1^

A prova do TEC consta de 120 questões de múltipla escolha, com cinco alternativas cada, sendo apenas uma correta. Há uma preocupação em relação ao grau de dificuldade das questões selecionadas para a prova e, dessa forma, a comissão classifica caracteriza-as como difíceis, médias e fáceis. No entanto, até o momento, essa classificação é feita de modo subjetivo, ou seja, de acordo com a opinião dos membros da CJTEC e não através de uma metodologia psicométrica, que avalia o grau de dificuldade para quem faz a prova. ^2^

A Teoria de Resposta ao Item (TRI), tem sido utilizada recentemente como avaliação psicométrica na análise e na interpretação dos resultados nos diversos cenários de provas e concursos. ^2^

Até o momento, não foi realizada nenhuma avaliação psicométrica do exame do TEC e, em virtude da importância da prova, é imprescindível conhecer se esse modelo de avaliação fornece realmente uma medida coerente e confiável do ponto de vista técnico. Diante do exposto, esse estudo teve como objetivo avaliar as propriedades psicométricas da prova do TEC no ano de 2019, em relação à TRI.

## Métodos

### Desenho do estudo

Foi um estudo observacional, com a análise psicométrica das 120 questões da prova realizada por 1120 candidatos, para a obtenção do TEC, no dia 27 de outubro de 2019, das 13:00 às 18:00 horas, em uma Universidade Privada de São Paulo.

### Critérios de inclusão e exclusão

Foram incluídos todos os gabaritos entregues pelos candidatos que realizaram a prova para obtenção do TEC em 2019. Foram excluídas duas questões após a fase recursal, além da prova de um candidato que respondeu apenas duas questões.

### Amostra

A Amostra, que teve duas questões excluídas na fase recursal, foi constituída por gabaritos de 118 questões, que foram respondidas e entregues pelos médicos que realizaram a prova para a obtenção do TEC no ano de 2019.

### Coleta de Dados

Os dados dos candidatos foram extraídos diretamente do banco de dados da empresa responsável pela elaboração da prova (Segmento Farma Editores Ltda., em parceria com a Simples Detalhe Assessoria, Planejamento e organização de Eventos Ltda. e a Picsis informática indústria e comércio Ltda.), e organizados em planilhas Microsoft Excel ^®^ .

A partir desses dados, foram geradas planilhas separadas para os dados de identificação e para os dados referentes às notas de cada prova. Os nomes dos candidatos foram excluídos das planilhas, com o objetivo de manter o sigilo da pesquisa, sendo a identificação de cada candidato feita por um número.

### Aspectos éticos

Foram utilizadas bases de dados secundárias, sem identificação dos participantes, de modo que não houve necessidade de utilização de Termo de Consentimento Livre e Esclarecido (TCLE). No entanto, para a obtenção do banco de dados, foi firmado um Termo de Consentimento de Utilização do Banco de Dados (TCUD), o qual foi encaminhado, inicialmente, para a SBC e, posteriormente, ao Comitê de Ética em Pesquisa (CEP), com número do parecer: 4.030.702.

### Análise estatística

Realizamos uma avaliação psicométrica da prova aplicada aos candidatos ao TEC em 2019 pela SBC, utilizando a TRI. A TRI busca determinar o nível de aptidão do candidato (traço latente, parâmetro *teta* (θ)] e a probabilidade de um indivíduo com certo nível de aptidão em responder acertadamente os itens conforme o grau de dificuldade.

Para análise do traço latente, a TRI avalia os seguintes parâmetros:

- Item Discriminação ( *a* ): consiste na avaliação da aptidão do item em distinguir indivíduos com habilidades diferentes;
- Item Dificuldade ( *b* ): trata da habilidade mínima que um respondente precisa para ter uma grande probabilidade de dar a resposta correta;
- Acerto ao Acaso ( *c* ): a probabilidade de um respondente com baixa proficiência responder corretamente um item.

Desta forma, a TRI procura medir variáveis não observáveis (traço latente) que possam influenciar as respostas dadas aos itens, utilizando a aferição das variáveis observáveis (respostas aos itens), e estabelecendo uma relação entre a habilidade do respondente e os parâmetros do item com a probabilidade de acerto ao item. Assim, quanto maior a aptidão de um candidato, maior será sua probabilidade de responder corretamente o item no instrumento de avaliação.

Duas importantes suposições da TRI são a Unidimensionalidade que postula que há apenas uma aptidão (θ) responsável pela realização de um conjunto de itens de um teste, e a Independência local, que implica que o desempenho do sujeito em um item não afeta o desempenho em outro, pois cada item é respondido em função da aptidão dominante (θ) do candidato para aquele item.

O modelo estatístico da TRI predominantemente utilizado no Brasil é o modelo Logístico unidimensional de três parâmetros. Os modelos Logísticos unidimensional com um e dois parâmetros não são convenientes na análise do presente estudo uma vez que, pelos resultados obtidos no modelo de três parâmetros, o *acerto ao acaso* variou muito entre os 120 itens da prova aplicada em 2019.

### Metodologia de cálculos da TRI

Modelo logístico Unidimensional da TRI com 3 parâmetros


$$P\left(U_{ij}=1|\theta_{j}\right)=c_{i}+\left(1-c_{i}\right)\frac{1}{1+e^{-Da_{i}\left(\theta_{j}-b_{i}\right)}}$$

com i = 1, 2, ..., I e j = 1, 2, ..., n, onde:

- **U**_**ij**_ é uma variável dicotômica que assume os valores 1, quando o indivíduo j responde corretamente o item i, ou 0 quando o indivíduo j não responde corretamente ao item i.
- **θ**_**j**_ representa a habilidade (traço latente) do j-ésimo indivíduo.
- **P(U**_**ij**_ **=1** |θ _**j**_ **)** é a probabilidade de um indivíduo j com habilidade θj responder corretamente o item i e é chamada de Função de Resposta do Item – FRI.
- **b**_**i**_ é o parâmetro de dificuldade (ou de posição) do item i, medido na mesma escala da habilidade.
- **a**_**i**_ é o parâmetro de discriminação (ou de inclinação) do item i, com valor proporcional à inclinação da Curva Característica do Item — CCI no ponto bi.
- **c**_**i**_ é o parâmetro do item que representa a probabilidade de indivíduos com baixa habilidade responderem corretamente o item i (muitas vezes referido como a probabilidade de acerto casual).
- **D** é um fator de escala, constante e igual a 1.

Os valores dos parâmetros *a, b e c* , são calculados por meio de pré-testagens (calibragem) dos itens, utilizando o método da máxima verossimilhança ( **L** , de *likelihood* ), que trabalha com as derivadas. Sua fórmula é a seguinte:


$$L\left(u_{1s^{\prime }}u_{2s^{s^{\prime }}}\ldots ,u_{ns}|\theta \right)=\overset{n}{\underset{i=1}{\prod }}\;Pi(\theta s{)}^{u_{si}}Qi(\theta s{)}^{1-u_{si}}$$

Em que:

- **i** = 1, 2, ..., n itens
- **u**_**is**_ = resposta do sujeito a cada item (1 = acertou, 0 = errou)

Para se calcular a aptidão/proficiência de um candidato, temos que achar o máximo da função acima. Primeiramente, identifica-se a probabilidade de acerto [(Pi(θ)] de cada um dos itens do teste utilizando um dos três modelos da TRI (1PL, 2PL, 3PL – na presente pesquisa foi utilizado o modelo de três parâmetros – 3PL). Posteriormente, de forma empírica, substituem-se os valores de θ numa faixa de - 5 a + 5 (-5,00 ≤ θ ≤ +5,00, normalmente, utiliza-se -3,00 ≤ θ ≤ +3,00) ou emprega-se o algoritmo de iteração de Newton-Raphson para se calcular o máximo da função L. Esse máximo, com base nos valores de θ, é a aptidão/proficiência do candidato obtido no teste aplicado.

### Curva Característica do Item (CCI)

O modelo matemático que define a TRI é uma função de probabilidade. Portanto, sua imagem estará sempre no intervalo [0,1]. O número U _ij_ =1|θ _j_ ) pode ser identificado pela proporção de respostas corretas ao item i no grupo de indivíduos com habilidade θj. Essa relação é descrita por uma curva sigmoide, em que o eixo horizontal representa a escala de aptidão e o eixo vertical a probabilidade do indivíduo com uma habilidade θj dar a resposta correta ao item i. Podemos destacar duas assíntotas horizontais e notam-se, com uma certa precisão, os três parâmetros do item.

### Curva de Informação – I(θ)

Precisão da informação significa a exatidão que um item representa aquilo que ele pretende medir. Neste contexto, precisão significa o quão bem o item prediz o critério ou representa o traço latente (θ). Assim, a função de informação da TRI segue o cálculo do erro de estimação, isto é, o quanto o escore obtido pelo sujeito num teste se afasta do seu escore verdadeiro. O próprio conceito de função de informação é o recíproco da variância, ou seja: I = 1 / S ^2^ . A função de informação corresponde ao conceito de carga fatorial do item da análise fatorial, na visão do modelo do traço latente, pois a carga fatorial representa a covariância entre o item (representação comportamental) e o traço latente (teta). A curva de informação do teste mostra a quantidade de informação fornecida pelo teste a um certo nível teta; apresenta a amplitude do teta para a qual o teste fornece informação confiável, dizendo que fora dessa amplitude o teste produz mais informação errônea (erro) sobre o teta que informação correta. Assim, a curva de informação tem interface com ambos os parâmetros dos testes, isto é, validade e precisão, mas não se confunde com nenhum dos dois. A representação dessa informação do item assemelha-se a uma curva de tipo normal (em forma de “sino”).

Na presente análise, foi adotado o critério de, pelo menos, 25% de “acerto ao acaso” como insatisfatório para um determinado item da prova. Assim, como foram aplicadas 1120 provas, 5% a mais de “acerto ao acaso” além do esperado (20%) é considerado muito alto e, portanto, o item avaliado apresenta algum problema em sua formulação ou nas opções de respostas. O “acerto ao acaso” é demonstrável pela falta de coerência do candidato, em errar itens fáceis e, de modo contraditório, acertar itens difíceis, teoricamente sem proficiência para tal.

## Resultados

Os resultados apresentados referem-se à análise psicométrica dos 118 itens da prova aplicada aos candidatos ao TEC em 2019, utilizando um modelo logístico unidimensional de três parâmetros da TRI: discriminação (a), dificuldade (b) e acerto ao acaso (c).

Durante a análise, verificou-se que um item (Questão nº 110) apresentou nível negativo para o parâmetro *discriminação* ( *a* = - 0,174), sugerindo que quanto maior o nível de conhecimento do candidato menor a chance de acertar o item, um resultado incoerente com o objetivo do parâmetro, por essa razão, esse item não foi incluído no modelo da análise final.

A Tabela 1 apresenta a distribuição dos 118 itens da prova em relação ao parâmetro *discriminação* . Observa-se que 18,7% desses apresentavam “muito baixo” ou “baixo” poder de *discriminação* (a ≤ 0,65); 49,1% “moderada” *discriminação* (0,651 < a ≤ 1,350) e 32,2% dos itens apresentavam “alto” ou “muito alto” poder de *discriminação* (a ≥ 1,351).

**Tabela 1:** Distribuição dos itens da prova em relação ao parâmetro *discriminação* da Teoria de Resposta ao Item (TRI)

Classificação do poder de discriminação (a)	Frequência (n)	%
≤ 0,35 (muito baixa)	12	10,2
De 0,351 a 0,650 (baixa)	10	8,5
De 0,651 a 1,350 (moderada)	58	49,1
De 1,351 a 1,700 (alta)	25	21,2
> 1,700 (muito alta)	13	11,0
Total	118	100,0

Base de Dados: 1.120 candidatos. Nota: 2 itens da prova anulados (itens 23 e 46).

A Tabela 2 apresenta a distribuição dos 118 itens da prova em relação ao parâmetro *dificuldade* . Observa-se que 49,2% desses foram classificados como “fácil” ou “muito fácil” ( *b* < -0,52); 22,0% classificados com *dificuldade* moderada (-0,51 ≤ *b* ≤ 0,51); e 28,8% dos itens foram classificados como difícil ou muito difícil ( *b* ≥ 0,52).

**Tabela 2:** Distribuição dos itens da prova em relação ao parâmetro *dificuldade* pela TRI

Classificação do parâmetro dificuldade (b)	Frequência (n)	%
≤ -1,28 (Muito fácil)	31	26,3
De -1,27 a - 0,52 (Fácil)	27	22,9
De -0,51 a 0,51 (Moderada)	26	22,0
De 0,52 a 1,27 (Difícil)	19	16,1
≥ 1,28 (Muito difícil)	15	12,7
Total	118	100,0

Fonte: Elaborada pelos autores; Base de Dados: 1.120 candidatos. Nota: 2 itens da prova anulados (itens 23 e 46).

A Tabela 3 apresenta a distribuição dos 118 itens da prova em relação ao parâmetro *acerto ao acaso* , onde observa-se que 41,5% dos itens apresentaram alta probabilidade de *acerto ao acaso* , conforme a metodologia da TRI.

**Tabela 3:** Distribuição dos itens da prova em relação aos percentuais de *acertos ao acaso* pela teoria de resposta ao item

Percentual de acertos ao acaso (c)	Frequência (n)	%
≤ 10,0%	48	40,7
De 10,1 a 25,0%	21	17,8
De 25,1 a 40,0%	20	16,9
De 40,1 a 60,0%	19	16,1
> 60,0%	10	8,5
Total	118	100,0

Fonte: Elaborada pelos autores; Base de Dados: 1.120 candidatos. Nota: 2 itens da prova anulados (nos 23 e 46).

Sobre a CCI, 58,5% dos itens foram considerados insatisfatórios. Já em relação a curva de informação, 78.8% dos itens foram satisfatórios ( Tabela 4 ).

**Tabela 4:** Distribuição dos itens da prova segundo classificação (satisfatória ou insatisfatória) da Curva Característica do Item e da Curva de Informação da teoria de resposta ao item

Curva Característica do Item	Frequência (n)	%
Satisfatória	49	41,5
Insatisfatória	69	58,5
**Curva de Informação**	**Frequência (n)**	**%**
Satisfatória	93	78,8
Insatisfatória	25	21,2

Fonte: Elaborada pelos autores; Base de Dados: 1.120 candidatos. Nota: 2 itens da prova anulados (nos 23 e 46).

A análise individual dos itens da prova pela TRI identificou 69 itens que apresentavam algum tipo de problema em relação aos três parâmetros e, portanto, considerados com baixo poder de gerar informação em relação à identificação do traço latente (θ), que avalia a proficiência do candidato. Assim, os 49 itens restantes da prova foram analisados pela TRI e comparados ao modelo inicial da prova com 118 itens.

**Figura 1:**
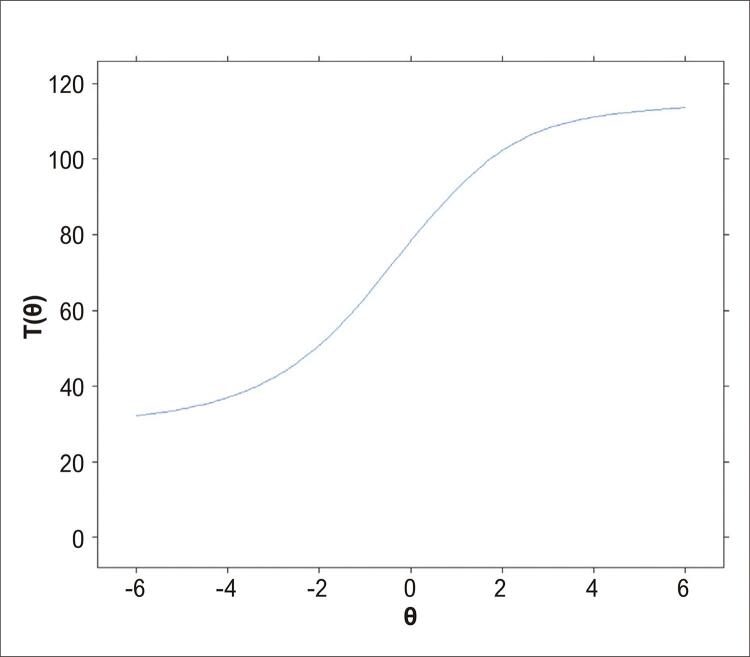
Nota: T(θ) – estimada pela teoria de resposta ao item considerando-se os 118 itens da prova, para cada candidato de acordo com sua aptidão (θ).

A Figura 1 apresenta a CCI considerando os 118 itens da prova pelo modelo da TRI. O resultado mostra que quanto maior a aptidão (θ) do candidato, maior será o número de itens com resposta correta. Um candidato com aptidão igual a 0 (θ = 0 – aptidão mediana, θ entre -1 e +1) é esperado que acerte, aproximadamente, 80 dos 118 itens da prova (67,8%). Além disso, um candidato com muito baixo nível de aptidão (θ < - 4,0) é esperado que acerte pelo menos 36 dos 118 itens da prova (30,5%).

**Figura 2:**
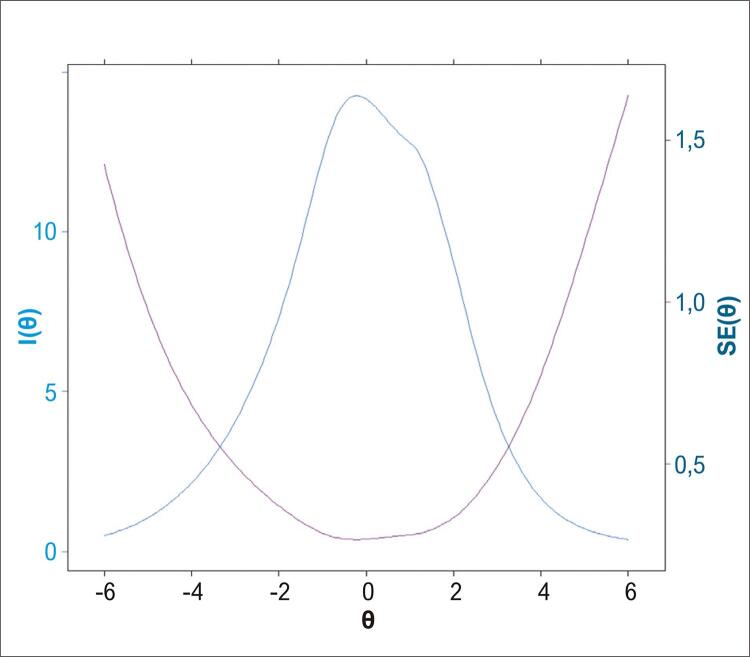
Curva de Informação: I(θ) – e erro padrão gerados pela teoria de resposta ao item considerando os 118 itens da prova.

A *Curva de Informação* ( Figura 2 ) para o conjunto dos 118 itens da prova mostra que a quantidade máxima de informação recuperada sobre o raciocínio analógico do candidato encontra-se em torno da mediana da aptidão, ou seja, valor de θ próximo a 0. Além disso, para os valores extremos de θ, a prova produz mais erro de informação do que informação legítima, e nos valores de θ entre -3,2 a +3,1 encontra-se o máximo de informação gerado pela avaliação.

**Figura 3:**
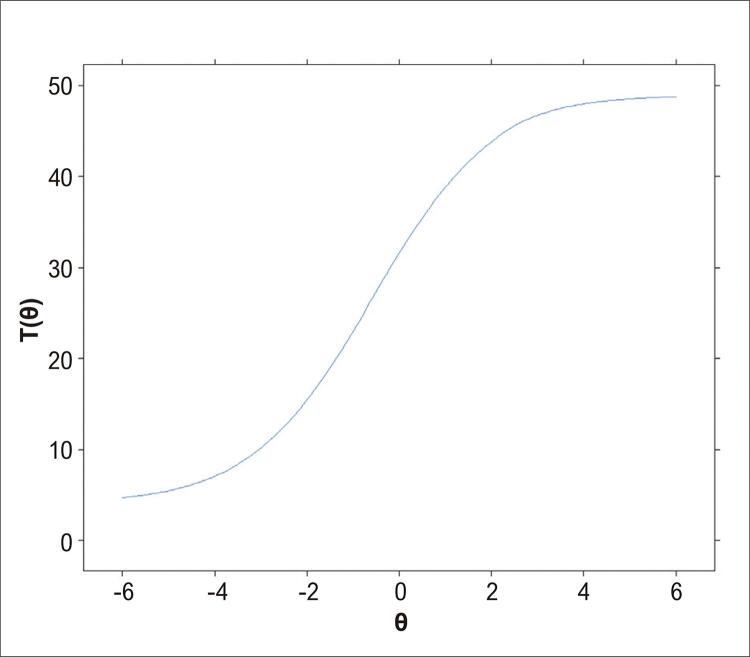
Nota: T(θ) – estimada pela teoria de resposta o item considerando os 49 itens da prova, para cada candidato de acordo com sua aptidão (θ).

A Figura 3 mostra a CCI para o conjunto dos 49 itens da prova que restaram após a extração dos itens com problemas nos parâmetros da TRI. O resultado mostra que quanto maior a aptidão (θ) do candidato, maior será o número de itens com resposta correta. Assim, é esperado que um candidato com aptidão igual a 0 (θ = 0 – aptidão mediana, θ entre -1 e +1) acerte, aproximadamente, 32 dos 49 itens da prova (65,3%), e um candidato com nível de aptidão muito baixo (θ < -4,0) acerte pelo menos quatro dos 49 itens da prova (8,2%). Portanto, considerando os dados da TRI para os 49 itens, os candidatos precisarão de um nível aptidão (θ) maior em relação à exigida para os 118 itens da prova.

**Figura 4:**
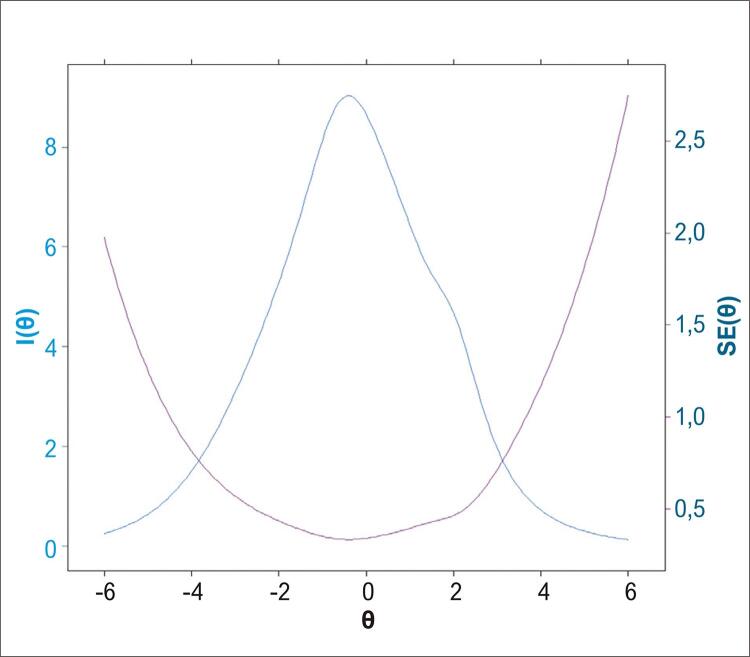
Curva de Informação: I(θ) – e erro padrão gerados pela teoria de resposta ao item considerando os 49 itens da prova.

A *Curva de Informação* ( Figura 4 ) para os 49 itens da prova mostra que a quantidade de informação máxima recuperada sobre o raciocínio analógico do candidato encontra-se, também, em torno da mediana da aptidão, ou seja, valor de θ próximo de 0 (zero). Além disso, para os valores extremos dos níveis de θ, a prova produz mais erro de informação do que informação legítima, sendo que para valores de θ variando entre -4,0 a +3,2 encontra-se o máximo de informação gerado pela avaliação.

**Figura 5:**
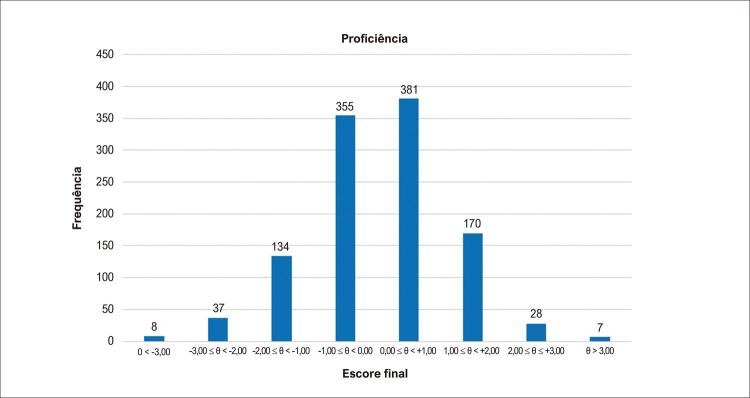
Resultado da proficiência gerados pela TRI.

A Figura 5 mostra resultado da proficiência gerado pela TRI, considerando-se os 49 itens excluídos da prova inicialmente aplicada. Nota-se uma curva típica de Gauss, que mostra o nível médio de proficiência dos candidatos com um padrão normal de distribuição.

Fonte: Elaborado pelos autores.

## Discussão

O objetivo do presente estudo foi avaliar os itens da prova para obtenção do TEC em 2019, em relação aos seus parâmetros psicométricos pela TRI. Até então, o único parâmetro conhecido pela CJTEC era o grau de dificuldade dos itens, julgados como fáceis, médios ou difíceis, com base no conhecimento e na experiência dos integrantes da comissão. No entanto esta forma de avaliação é subjetiva e carece de validade.

Em relação ao parâmetro *discriminação* , observou-se que apenas 32,2% dos itens apresentavam poder de *discriminação* “alto” ou “muito alto”. Informação relevante, uma vez que a *discriminação* de um item relaciona-se à sua capacidade de identificar candidatos com proficiências distintas, por avaliar a probabilidade de indivíduos com diferentes proficiências responderem corretamente o item. Fatos semelhantes foram observados nas provas do Exame Nacional de Desempenho dos Estudantes (ENADE), nos anos de 2010, 2011 e 2012, que ao serem submetidas à análise psicométrica pôde-se identificar questões com baixo poder de discriminação, contribuindo tecnicamente para melhorias na elaboração futura de novos itens para as provas do ENADE. ^3 , 4^

A respeito do parâmetro *dificuldade* , observou-se que 49,2% dos itens da prova, eram categorizados pela TRI como “fácil” ou “muito fácil” e apenas 22% como de “moderada” dificuldade. Isso denota um desbalanceamento da prova em relação à psicometria, que recomenda a seguinte proporção de facilidade dos itens: muito fáceis (10%), fáceis (20%), moderados (40%), difíceis (20%) e muito difíceis (10%). ^4^ A proporção de itens “difíceis e “muito difíceis” se encontrava adequada. Chama a atenção que a prova do TEC em 2019 apresentava predominantemente itens considerados “fáceis”.

Sobre o parâmetro *acerto ao acaso* , constatou-se que 41,5% dos itens da prova do TEC apresentavam grande possibilidade de *acerto ao acaso* , um percentual elevado para uma avaliação certificativa do porte da prova do TEC. A CCI foi insatisfatória para 58,5% dos itens e a *curva de informação* foi satisfatória em 78,8% dos itens, o que demonstra que o acerto aos itens não apresentava boa correlação à proficiência do candidato, apesar de ser capaz de medir o traço latente.

Ao proceder a análise individual dos itens da prova, constatou-se que 69 itens apresentavam problemas em relação aos parâmetros da TRI avaliados e, portanto, com baixo poder de informação em relação à identificação do traço latente dos candidatos. Apesar disso, a CCI era coerente em relação à aptidão do candidato e seu número de acertos aos itens, ou seja, quanto maior a aptidão do candidato, maior o número de itens corretos. Porém, a CCI demonstrou que candidatos com nível de aptidão baixa eram capazes de acertar até 30,5% dos itens da prova. Em relação à identificação de itens deficientes, o resultado foi semelhante ao observado na prova da Olimpíada Brasileira de Matemática das Escolas Públicas em 2016, na qual 11 de suas 20 questões eram deficientes em relação aos parâmetros da Teoria Clássica de Teste. ^3^

Ao retirar os itens com problemas nos parâmetros da TRI da prova original, restaram 49 itens, que ao serem avaliados em conjunto como um “modelo alternativo” de prova, mantiveram as mesmas características psicométricas da CCI da prova original e uma distribuição normal da média de proficiência dos candidatos. No entanto, este modelo reduziu de 30,5% para 8,2% o percentual de candidatos que mesmo com baixa proficiência acertariam os itens da prova. Essa redução significativa deve-se à diminuição do *acerto ao acaso* dos itens, resultado relevante observado nesse “modelo alternativo” de prova orientado pela TRI.

Nesse sentido, observa-se a importância da análise dos parâmetros psicométricos em uma prova, os quais apresentam medidas matemáticas, contribuindo para a construção de um exame composto por itens “calibrados”, e o aprimoramento do instrumento de avaliação.

Até onde se sabe, este é o primeiro estudo a avaliar as características psicométricas de uma prova de título de especialista da AMB e seus resultados contribuirão para reflexões e aprimoramentos desses instrumentos certificadores. Por esse motivo, não foram encontradas outras referências bibliográficas que permitissem comparar os resultados encontrados com os de outras sociedades de especialidades, mas há publicações disponíveis em outros cenários.

O presente estudo oportuniza a discussão sobre o modelo atual de confecção da prova do TEC. Nele, os itens são elaborados por um conjunto heterogêneo de pessoas, que não discutem a prova como um instrumento único, e as provas não têm as mesmas características psicométricas a cada ano, impossibilitando a comparabilidade no tempo.

Esses dados contribuem para que a CJTEC possa, inclusive, avaliar o número de questões que são necessárias na prova do TEC, uma vez que, pela TRI, um modelo ajustado com 49 itens apresentou os mesmos resultados certificadores. A possibilidade da redução do número de questões de uma prova, orientada por métodos psicométricos, pode produzir um instrumento de avaliação capaz de discriminar com maior precisão os candidatos merecedores do TEC, e de lhes oferecer um exame menos cansativo, favorecendo inclusive, um melhor desempenho dos candidatos. Assim, a probabilidade de concessão de títulos favorecida pelos *acertos ao acaso* seria menor, aprimorando-se a identificação dos participantes proficientes, com um padrão de respostas coerentes em relação aos parâmetros estudados.

Com base nos achados deste estudo e seguindo as tendências de outras instituições que já utilizam a TRI para a seleção dos itens de suas avaliações, ^4^ essa metodologia pode incrementar, de forma impactante, a qualidade das provas de títulos das diversas especialidades da AMB, contribuindo para a identificação de candidatos com as competências esperadas para o exercício de sua especialidade no Brasil.

Ao apoiar este estudo, a SBC demonstra o compromisso no aprimoramento do seu instrumento de certificação profissional, a prova do TEC. Os resultados deste estudo inédito são relevantes para o aprimoramento técnico da elaboração dos itens para a prova de título da SBC, e servirá de referencial para outras Sociedades de Especialidades da AMB.

### Limitações e perspectivas

O presente estudo apresenta algumas limitações. Para a obtenção de melhores resultados pela TRI, é desejável a construção de um banco de dados com itens previamente utilizados e calibrados. Isso não foi possível, uma vez que este é o primeiro estudo realizado com uma prova do TEC e, provavelmente, também de um exame para concessão do título de especialista da AMB. Outra limitação diz respeito ao banco de dados avaliado, pois embora tenhamos considerado a prova realizada no ano de 2019, todas as edições da prova do TEC foram independentes, apesar de seguirem a mesma metodologia de elaboração. Por isso, não é possível afirmar que os resultados aqui apresentados possam ser extrapolados para os concursos anteriormente realizados. Apesar disso, o estudo apresenta importantes contribuições para que a SBC e AMB possam aprimorar os instrumentos certificativos para concessão de títulos de especialistas.

## Conclusão

O presente estudo permitiu verificar as propriedades psicométricas da prova do TEC de 2019 usando a TRI. O exame apresentou um maior percentual de questões fáceis, com cerca de um terço de itens com alto poder de discriminação, e os demais necessitando melhorias na elaboração, uma vez que apresentaram elevada probabilidade de acerto ao acaso. O estudo sugere que uma avaliação com menor número de questões seria capaz de apresentar as mesmas características psicométricas da prova analisada, mas com um potencial de reduzir o acerto ao acaso dos itens. Os resultados deste trabalho contribuem para o aprimoramento da prova do TEC, importante instrumento que certifica o especialista em cardiologia no Brasil.
