# Supplementary material for: Avaliação Psicométrica da Prova de Título de Especialista em Cardiologia da Sociedade Brasileira de Cardiologia
Source: Arq Bras Cardiol. 2022 Nov 9;119(5 Suppl 1):6–13. [Article in Portuguese] doi: 10.36660/abc.20220355 (PMC9750200; doi:10.36660/abc.20220355)
Supplement: Translation

# Translation: Psychometric Evaluation of the Cardiology Certification Exam of the Brazilian Society of Cardiology

**Authors:** Gustavo Eugênio Martins Marinho, José Maria Peixoto, José Knopfholz, Marcus Vinicius Santos Andrade

**Affiliations:** 1 Brazil Faculdade de Medicina na Universidade José do Rosário Vellano (UNIFENAS), Alfenas, MG – Brazil; 2 Brazil Universidade José do Rosário Vellano (UNIFENAS), Belo Horizonte, MG – Brazil; 3 Brazil Programa de Mestrado Profissional em Ensino em Saúde da Universidade José do Rosário Vellano (UNIFENAS-BH), Belo Horizonte, MG – Brazil; 4 Brazil Pontifícia Universidade Católica do Paraná, Curitiba, PR – Brazil; 5 Brazil Programa de Especialização em Educação em Saúde da Universidade de São Paulo, São Paulo, SP – Brazil; 6 Brazil Escola Bahiana de Medicina, Salvador, BA – Brazil; 7 Brazil Hospital Santa Izabel - Santa Casa da Bahia, Salvador, BA – Brazil; 8 Brazil Hospital Aliança, Salvador, BA – Brazil

**Keywords:** Specialization, Cardiology, Psychometrics

## Abstract

**Background:**

The Cardiology Certification Exam is issued annually by the Brazilian Cardiology Society and set and applied by the Judging Committee for the Cardiologist Title (CJTEC). The psychometric analysis of the exam items using the Item Response Theory (IRT) may provide robust data that can help in the continuous improvement of this instrument.

**Objectives:**

To evaluate the psychometric properties of the 2019 Cardiology Certification Exam in relation to the IR parameters.

**Methods:**

This was an observational study, with psychometric analysis of the 120 questions of the exam taken by 1,120 candidates for the title of Cardiologist in 2019.

**Results:**

The IRT analysis revealed that 32.2% of the items had a “high” or “very high” discriminating power, 49.2% were categorized as “easy” or “very easy”, and 41.5% showed a high probability of a correct guessing. Sixty-nine deficient items in terms of the IRT parameters were identified, which were then considered poorly effective in evaluating the candidate’s ability.

**Conclusions:**

The psychometric analysis of the 2019 Cardiology Certification Exam by the IRT revealed a high percentage of easy questions, with nearly two thirds of the items with a high probability of correct guessing. These data may serve as a basis for a series of discussions and proposals for the elaboration of future certificate exams in Cardiology.

## Introduction

The title of specialist has become a constant goal among Brazilian physicians. The reasons range from knowledge gain, prerequisite to participate in public calls, to becoming a member of medical cooperatives in the labor market, evidencing that medical titles enhance both professional status and the prestige of the specialty.

The Cardiology Certification Exam (CCE) has been issued by the Brazilian Cardiology Society (SBC) since 1968, but was legalized only in 1989 by the Brazilian Medical Association (AMB) and the Federal Council of Medicine (CFM) by the 1286/89 resolution. In this context, in 1992, the Judging Committee for the Cardiologist title (CJTEC) was created.^1^

The CCE consists of 120 multiple-choice questions with five choices with one correct answer each. There is a concern regarding the difficulty level of the questions, and in this respect, the CJTEC classify them as highly, moderately or little difficult. However, this classification has been done subjectively, *i.e* ., according to the opinion of the CJTEC members, without the use of a psychometric methodology that evaluates the degree of difficulty faced by the applicants.^2^

The item response theory (IRT) has been recently used as a psychometric method for the analysis and interpretation of the results in different scenarios of exams and public calls.^2^

So far, the CCE has not undergone a psychometric test, and considering the importance of this exam, it is essential to know whether this method of evaluation provides a reliable and coherent measure from the technical point of view. Based on this, this study aimed to assess the psychometric properties of the 2019 CCE in relation to the IRT.

## Methods

### Study design

This was an observational study, with psychometric analysis of 120 questions of the CCE taken by 1,120 applicants to obtain the title of cardiologist. The CCE was administered on October 27, 2019, from 13h to 18h at the Universidade Privada de São Paulo.

### Inclusion and exclusion criteria

All the answer keys delivered by the candidates who applied for the CCE in 2019 were included. After the appealing phase, two questions and one exam from an applicant who answered only two questions of the test were excluded.

### Sample

After the exclusion of two questions in the appealing phase, the sample consisted of answer keys of 118 questions, answered by physicians who applied for the CCE in 2019.

### Data Collection

Data were collected from the database of the agency responsible for elaborating the exam (Segmento Farma Editores Ltda., with the help of Simples Detalhe Assessoria, Planejamento e organização de Eventos Ltda. and Picsis informática indústria e comércio Ltda.) and plotted in Excel spreadsheets.

Separate spreadsheets were then generated, with identification data and exam scores. The names of the candidates were deleted from the spreadsheets for the sake of confidentiality, and the applicants were identified by numbers.

### Ethical aspects

Informed consent was waived since secondary databases were used, *i.e.* without participants’ identification. However, to construct the database, a consent form for the use of the data was signed, which was first sent to the SBC and then to the ethics committee (approval number 4.030.702).

### Statistical analysis

We performed a psychometric assessment of the 2019 CCE, offered by the SBC, using the IRT. The IRT aims to determine the applicant’s ability level (latent trait, theta [θ]), and the probability that a person with a given ability level will answer correctly a set of items according to their difficulty level.

For analysis of the latent trait, the IRT assesses the following parameters:

- Item Discrimination (a): performance of the item in differentiating between individuals possessing different levels of ability;
- Item Difficulty (b): minimum ability that a respondent must possess to be very likely to answer correctly;
- Guessing (ci): probability of a low-proficient respondent answering correctly an item.

Therefore, the IRT attempts to measure unobservable variables (latent trait) that may influence the answers given to the items, by measuring observed variables (responses). Thus, IRT establishes a relationship or the respondent’s ability and the item parameters with the probability of endorsing the correct answer for an item. The higher the person’s ability, the higher the respondent’s probability of answering correctly the instrument’s items.

Two important assumptions of the IRT are Unidimensionality, that assumes that there is only one latent trait (θ) affecting the responses observed for the items in the measure, and Local Independence, that assumes that the individual’s performance in separate items is mutually independent, since each answer is given according to the dominant ability (θ) to that item.

In Brazil, the most widely used IRT model is the unidimensional three-parameter logistic model. The unidimensional models with one or two parameters are not suitable for the analysis in the present study, since the results obtained from the three-parameter model revealed a great variation in the guessing item between the 120 questions of the exam applied in 2019.

IRT calculation methods:

Unidimensional three-parameter logistic model


$$P\left(U_{ij}=1|\theta_{j}\right)=c_{i}+\left(1-c_{i}\right)\frac{1}{1+e^{-Da_{i}\left(\theta_{j}-b_{i}\right)}}$$

with i = 1, 2, ..., I and j = 1, 2, ..., n, where:

- **U**_**ij**_ is a dichotomous variable that corresponds to 1, when the respondent j answers correctly the item i, or 0 when the respondent does not answer the item i correctly.
- **θ**_**j**_ represents the ability (latent trait) of the respondent number j.
- **P(U**_**ij**_ **=1** | θ_**j**_ **)** is the probability of the individual j with a θj ability to answer correctly the item i, and is called Item Response Function (IRF).
- **b**_**i**_ is the difficulty (or position) parameter, measured on the same scale as ability.
- **a**_**i**_ is the discrimination (or inclination) parameter of the item i, which is proportional to the inclination of the item characteristic curve (ICC) in the point bi
- **c**_**i**_ is the parameter that represents the probability of low-ability individuals answering correctly the item i by chance (often referred as the correct guessing probability)
- **D** is a scale factor, constant (=1).

Values of the *a, b* and *c* parameters are calculated by pre-testing (calibration) using the maximum likelihood ( **L** ) method, which works with derivatives and is defined as:


$$L\left(u_{1s^{\prime }}u_{2s^{\prime }}\ldots ,u_{ns}|\theta \right)=\overset{n}{\underset{i=1}{\prod }}\;\mathrm{Pi}(\theta s{)}^{u_{si}}Qi(\theta s{)}^{1-u_{si}}$$

The maximum likelihood ( **L** ) works with derivatives.

Where:

- **i** = 1, 2, ..., n items
- **u**_**is**_ = response of the individual to each item (1 = correct, 0 = wrong)

To calculate the ability/ proficiency of the applicant, we have first to determine the maximum value of the function above. First, the probability of correct responses [(Pi(θ)] of each item is determined using one of the three IRT models – 1PL, 2PL or 3PL. In the present study, the three-parameter model (3PL) was used. Then, θ is empirically substituted with values ranging from -5 to +5 (-5,00 ≤ θ ≤ +5,00, usually -3,00 ≤ θ ≤ +3,00), or the Newton-Raphson iteration algorithm is used to calculate the maximum of the L function. Based on the θ, this maximum represents the applicant’s ability/proficiency.

### Item characteristic curve (ICC)

The mathematical model that defines IRT is a probability function. Therefore, it will always be visualized within the interval [0,1]. The number U_ij_=1|θ_j_) can be identified by the proportion of correct answers to the item I in the group of individuals with ability θj. This ability is described as a sigmoid curve, where the horizontal axis represents the ability level and the vertical axis the probability of the individual with ability θj to give a correct response to the item i. Two horizontal asymptotes can be highlighted, and three parameters can be seen with some accuracy.

### Item information curve – I(θ)

Informatics accuracy is the degree of accuracy in which the item represents what it intends to measure. In this context, accuracy means how well the item predicts the criterion or represents the latent trait (θ). Thus, the IRT information function follows the calculation of the estimation error, that is, how much the score obtained by an individual in a test differs from the real score. The concept of information function itself is the reciprocal of variance, *i.e* ., I = 1/S^2^. The information function corresponds to the concept of factorial load of the item of the factorial analysis, from the latent trait model perspective, since the factorial load represents the covariance between the item (behavioral representation) and the latent trait (theta). The test information curve depicts the amount of information yielded by the test at any ability level; it presents the amplitude of theta to which the test provides reliable information, and out of which the test provides more erroneous than correct information about theta. Thus, the information curve has an interface to both test parameters, i.e., validity and accuracy, but is not cofounded by any of them. Representation of the information item resemble a normal-type (bell-shape) curve.

In the present analysis, a rate of correct guessing ≥ 25% in an item of the exam was considered unsatisfactory. Then, of the 1,120 exams, 5% of correct guessing higher than the expected rate (20%) is considered very high, and thus the item evaluated has some problem in its formulation or in the answer choices. The correct guessing can be seen by the lack of coherence of the candidate in answering incorrectly easy questions or, in contrast, answering correctly difficult questions, with no ability for it.

## Results

We present the results obtained from the psychometric analysis of 118 items of the exam the candidates applying for the CCE in 2019, using a three-parameter unidimensional logistic model of IRT: discrimination (a), difficulty (b) and guessing (c).

In the analysis, one item (question number 110) revealed a negative level for the *discrimination* parameter ( *a* = - 0.174), suggesting that the higher the respondent’s knowledge level, the lower the probability of correct answer, which is inconsistent with the objective of the parameter. For this reason, this item was not included in the final analysis.

Table 1 presents the distribution of the 118 items of the exam by their discriminating power. Of these items, 18.7% showed a very low or low discriminating power (a ≤ 0.65); 49.1% showed moderate discriminating power (0.651 < a ≤ 1.350) and 32.2% showed high or very high discriminating power (a ≥ 1.351).

**Table 1:** Distribution of the exam items by the item response theory (IRT) *discrimination* parameter

Classification of the discriminating power (a)	Frequency (n)	%
≤ 0,35 (very low)	12	10.2
0.351 - 0.650 (low)	10	8.5
0.651 - 1.350 (moderate)	58	49.1
1.351 - 1.700 (high)	25	21.2
> 1.700 (very high)	13	11.0
Total	118	100.0

Database: 1,120 candidates. Note: Two items cancelled (items 23 and 46)

Table 2 presents the distribution of the 118 items of the exam according to the *difficulty* parameter. Of these items, 49.2% were classified as easy or very easy (b < -0,52); 22.0% were moderately difficult (-0.51 ≤ b ≤ 0.51); and 28.8% were classified as difficult or very difficult (b ≥ 0.52).

**Table 2:** Distribution of the exam items by the item response theory (IRT) *difficulty* parameter

Classification of the difficulty parameter (b)	Frequency (n)	%
≤ -1.28 (very easy)	31	26.3
-1.27 – -0.52 (easy)	27	22.9
-0.51 - 0.51 (moderate)	26	22.0
0.52 – 1.27 (difficult)	19	16.1
≥ 1.28 (very difficult)	15	12.7
Total	118	100.0

Source: The authors; database: 1,120 candidates. Note: Two items cancelled (items 23 and 46).

Table 3 presents the distribution of the 118 items of the exam according to the *guessing* parameter. Of these, 41.5% of the items showed a high probability of guessing correctly according to the IRT methodology.

**Table 3:** Distribution of the exam items by the percentage of *correct guessing* according to the item response theory (IRT)

Percentage of correct guessing (c)	Frequency (n)	%
≤ 10.0%	48	40.7
10.1 - 25.0%	21	17.8
25.1 - 40.0%	20	16.9
40.1 - 60.0%	19	16.1
> 60.0%	10	8.5
Total	118	100.0

Source: The authors; database: 1,120 candidates. Note: Two items cancelled (items 23 and 46).

According to the ICC and the information curve, 58.5% and 78.8% of the items, respectively, were considered unsatisfactory ( Table 4 ).

**Table 4:** Distribution of the exam items according to the item characteristic curve and the information curve of the item response theory

Item characteristic curve	Frequency (n)	%
Satisfactory	49	41.5
Unsatisfactory	69	58.5
**Information curve**	**Frequency (n)**	**%**
Satisfactory	93	78.8
Unsatisfactory	25	21.2

Source: The authors; database: 1,120 candidates. Note: Two items cancelled (items 23 and 46).

Individual analysis of the exam items by the IRT identified 69 deficient items in relation to the three parameters, that were then considered to have a low probability of providing information about the latent trait (θ), which evaluates the ability of the candidate. Thus, the other 49 items were analyzed by the IRT and compared with the initial model composed of 118 items.

Figure 1 shows the ICC considering the 118 items by the IRT method. The results showed that the higher the applicant’s ability (θ), the higher the number of correct answers. It is expected that a medium-ability respondent answers approximately 80 (out of 118, 67.8%) items correctly. In addition, a very low-ability candidate (θ < -4.0) is expected to answer at least 36 (out of 118, 30.5%) items correctly.

**Figure 1:** Score: T(θ) – of each respondent, estimated by the item response theory (IRT) considering a total of 118 exam items, according to the candidate’s ability (θ).

The information curve ( Figure 2 ) for the 118 items showed that the maximum amount of information about the logical reasoning of the candidate was near the median ability, *i.e* ., θ near zero. Besides, for the extreme values of θ, the exam produces more information error than legitimate information, and the maximum information generated by the exam is within θ values between -3.2 and +3.1.

**Figure 2:** Information curve: I(θ) – and standard error of each candidate, generated by the item response theory, according to the respondent’s ability (θ).

Figure 3 shows the ICC for the 49 items remaining after the items with problems related to the IRT were excluded. The result shows that the higher the ability (θ) the higher the number of correct responses. Thus, it is expected that a 0-ability candidate (θ = 0 – median ability, -1 < θ < +1) answers approximately 32 questions (out of 49, 65.3%) correctly, and a very low-ability candidate (θ < -4.0) answers at least four (out of 49, 8.2%) correctly. Therefore, considering the IRT data for the 49 items, the candidates will require a higher ability level (θ) than that required for the 118 exam items.

**Figure 3:** Score: T(θ) – of each respondent, estimated by the item response theory (IRT) considering a total of 118 exam items, according to the candidate’s ability (θ).

The information curve ( Figure 4 ) for the 49 items showed that the maximum amount of information about the logical reasoning of the candidate was also near the median ability, i.e., θ near zero. Besides, for the extreme values of θ, the exam produces more information error than legitimate information, and the maximum information generated by the exam is within θ values between -4.0 and +3.2.

**Figure 4:** Information curve: I(θ) – and standard error generated by the item response theory, considering the 49 exam items.

Figure 5 depicts the results of ability generated by the IRT, considering the 49 items excluded from the exam initially applied. As can be seen, the mean ability level of the candidates shows a normal distribution, illustrated by a Gaussian pattern of data distribution.

**Figure 5:** Results of ability generated by the item response theory.

Source: The authors.

## Discussion

The aim of the present study was to analyze the items of the 2019 CCE regarding the psychometric parameters using the IRT. So far, the only known parameter was the degree of difficulty of the questions, categorized as easy, moderately difficult, or difficult, based on the knowledge and experience of the CJTEC members, who participated in the test formulation. However, this method of evaluation is subjective and lacks validity.

Regarding the *discrimination* parameter, only 32.2% of the items showed a “high” or “very high” discriminating power. This is a relevant information, since the discrimination of an item is related to its capacity to identify candidates with different ability levels, as the parameter measures the probability of individuals with different ability levels to answer an item correctly. Similar data were observed in the Brazilian National Exam for the Assessment of Student Performance (ENADE, *Exame Nacional de Desempenho dos Estudantes* ) applied in 2010, 2011 and 2012. Psychometric analysis of these exams identified several questions with low discriminating power, providing technical contributions for the formulation of new items for the following exams.^3 , 4^

With respect to the *difficulty* parameter, 49.2% of the items were categorized by the IRT as “easy” or “very easy”, and only 22% as “moderately difficult”. This indicates that the CCE was unbalanced in terms of psychometry, which recommends the following proportion of the items by difficulty level – very easy (10%), easy (20%), moderately difficulty (40%), difficult (20%) and very difficult (10%).^4^ The proportion of “difficult” and “very difficult” items was adequate. It is of note that the 2019 CCE was predominantly composed of “easy” items.

As for the *guessing* parameter, 41.5% of the CCE items had high probability of correct guessing. This is a high percentage considering the importance of the CCE. The ICC was unsatisfactory for 58.5% of the items and the information curve was satisfactory for 78.8% of the items, which indicates that answering correctly the items did not have a good correlation with the respondents’ ability, although it was able to measure the latent trait.

Individual analysis of the exam items identified 69 items with problems related to the IRT parameters and that were then considered to have a low probability of providing information about the candidates’ latent trait. Despite that, ICC was consistent regarding the candidate’s ability and the number of correct answers, *i.e* ., the higher the candidate’s ability, the higher the number of correct answers. Nevertheless, the ICC also revealed that low-ability respondents were able to answer up to 30.5% of the questions correctly. Similar result had been found in the 2016 Brazilian Mathematical Olympiad of Public Schools, in which 11 out of its 20 questions were deficient considering the classical test theory criteria.^3^

When the deficient items were removed from the original exam, the remaining 49 items were assessed as an “alternative model” of exam and maintained the same psychometric characteristics of the ICC of the original test and a normal distribution with the mean ability level of the candidates. However, with this model, the percentage of low-ability candidates who would answer the items correctly reduced from 30.5% to 8.2%. This significant reduction is attributed to a decrease in the percentage of correct guessing, which is a relevant result of the “alternative model” of exam, obtained by the IRT.

Therefore, psychometric parameters have mathematical measures, and their analysis in certification exams allows the improvement and construction of more “calibrated” instruments.

To the best of our knowledge, this is the first study to evaluate the psychometric characteristics of a specialist certification exam of the AMB, and the results will contribute to ideas and enhancement of this instrument. For this reason, we did not identify references of other medical societies or specialties to compare our results, although there are publications in other scenarios.

The present study opens the discussion about the current model of elaboration of the CCE. In this model, the items are constructed by a heterogeneous group of people, who do not discuss the exam as a unique instrument. Also, the annual exams do not have similar psychometric characteristics, which precludes their comparability over time.

In addition, our data contribute for the CJTEC to analyze the adequate number of questions of the CCE, since the IRT showed that an adjusted model of 49 items yielded the same certifying results. The possible reduction of the number of questions, when guided by psychometric methods, can produce an instrument able to discriminate, with greater accuracy, the candidates who are qualified for the title of cardiologist. Also, the exam would be less exhaustive, favoring a better performance of the candidates. Thus, the likelihood of passing the CCE due to a high percentage of correct answers by chance would be reduced, optimizing the identification of proficient professionals, able to give coherent answers in terms of the parameters evaluated.

Based on our findings and on the trends observed in other institutions where the IRT has been used for the selection of their exams’ items,^4^ this method can strongly impact the quality of the AMB specialty certification exams, contributing to the identification of candidates with the competencies expected for their practice.

The SBC supported this study, demonstrating its commitment in improving its professional certifying instrument, the CCE. The results of this unprecedented study are important for the technical improvement of the CCE items and will serve as a reference to other AMB specialty societies.

### Limitations and perspectives

The present study has some limitations. First, better results of the IRT can be obtained if a database with previously calibrated items is used. However, this was not possible in our study, since this is the first one to evaluate the CCE, and probably the first to evaluate an AMB medical specialty certificate examination. Another limitation is related to the database used in the study. Although we have analyzed the CCE applied in 2019, all previous editions were independent despite having been elaborated using the same method. Thus, we cannot affirm that the results obtained from the present study can be extrapolated to previous years’ editions. However, we do believe that the study provides important contributions for the SBC and the AMB to make improvements in their exams.

## Conclusion

This study allowed to determine the psychometric characteristics of the 2019 CCE by the IRT. The exam showed a high percentage of easy questions, with nearly one third of the questions with a high discriminating power and two thirds requiring improvements, as they had a high probability of correct guessing. The study suggests that an exam with a lower number of questions would show the same psychometric characteristics of the initial instrument, but with the potential to reduce the probability of guessing the answers correctly. These results contribute to the improvement of the CCE, an important certificate examination for the title of cardiologist in Brazil.
